# Clinical and Molecular Spectrum of Glutamate Dehydrogenase Gene Defects in 26 Chinese Congenital Hyperinsulinemia Patients

**DOI:** 10.1155/2018/2802540

**Published:** 2018-09-16

**Authors:** Chang Su, Xue-Jun Liang, Wen-Jing Li, Di Wu, Min Liu, Bing-Yan Cao, Jia-Jia Chen, Miao Qin, Xi Meng, Chun-Xiu Gong

**Affiliations:** Department of Pediatric Endocrinology, Genetic and Metabolism, Beijing Children's Hospital, Capital Medical University, National Center for Children's Health, Beijing 100045, China

## Abstract

**Objective:**

To characterize the genotype and phenotype of Chinese patients with congenital hyperinsulinism (CHI) caused by activating mutations in *GLUD1*, the gene that encodes mitochondrial enzyme glutamate dehydrogenase (GDH).

**Methods:**

The clinical data of glutamate dehydrogenase hyperinsulinism (GDH-HI) patients were reviewed, and gene mutations were confirmed by whole exome sequencing (WES) and Sanger DNA sequencing.

**Results:**

Twenty-six patients with GDH-HI heterozygous missense mutations were identified from 240 patients diagnosed as congenital hyperinsulinism over past 15 years. The median age at onset was 8 months (range: 1 day of life to 3 years). Seizure disorder was common in our cohort of patients (23/26). Four patients had normal serum ammonia levels; the median serum concentration was 101 *μ*mol/L (range: 37–190 *μ*mol/L). Hypoglycemic symptoms could be triggered by fasting or protein meals in all patients while blood glucose could be well controlled in all patients with diazoxide. Dosage of diazoxide could be reduced by protein restriction. Attempts to lower ammonia levels failed with different therapies such as protein restriction, benzoate, or N-carbamoyl glutamate. In follow-up, 15 of 26 patients had normal intelligence. Eleven patients developed epilepsy at the age of 6 months to 11 years. De novo mutations in *GLUD1* were found in 24 cases, and dominant inheritance was observed in the other two; all were heterozygous. A total of 35% (9/26) patients carried c.1493C>T (p.S445L) mutation.

**Conclusions:**

Phenotypic heterogeneity of GDH-HI patients was observed within the Chinese cohort in the present study. The fact that most patients had a *GLUD1* p. S445L mutation implies that this site could be a hotspot in Chinese patients. A high frequency of GDH-HI with normal ammonia has been reported in this study. Hence, *GLUD1* mutational analysis may be an important method to differential diagnosis of GDH-HI from other diazoxide-responsive CHI in Chinese patients.

## 1. Introduction

GDH-HI (hyperinsulinism hyperammonemia syndrome; HI/HA syndrome) caused by *GLUD1*-activating mutations is the second most common cause of congenital hyperinsulinism (CHI MIM256450), which is a genetic and phenotypic heterogeneous group of disorders associated with dysregulated insulin secretion [1]. The mitochondrial matrix enzyme glutamate dehydrogenase (GDH, EC 1.4.1.3), encoded by *GLUD1*, catalyzes the reversible reaction *α*-ketoglutarate + NH3 + NADH ↔ glutamate + NAD. In pancreatic *β*-cells, GDH participates in glucose-stimulated insulin secretion via glutamate formation, which is important for the amplifying pathway of insulin secretion. GDH activity is subject to complex regulation by both negative (GTP, palmitoyl-coenzyme A) and positive (ADP, leucine) allosteric effectors. In humans, GDH is predominantly expressed in the pancreatic islets, liver, kidney, and brain.

GDH-HI was first described in 1956 by Cochrane et al. [2]. Then, Stanley et al. [3] reported mutations in the *GLUD1* gene as the cause of this disorder in 1998. GDH-HI is characterized by both fasting and protein-sensitive hypoglycemia accompanied by persistently elevated plasma ammonia concentrations [3]. Medical management of GDH-HI disease relies on protein restriction and/or treatment with diazoxide. More than 100 patients with GDH-HI have been described [4]. Until now, nine Chinese GDH-HI patients have been reported in four different studies [5–8]. However, little is known about clinical characteristics, long-term follow-up results, and molecular spectrum of Chinese patients [5, 6, 8, 9]. In this study, we report 26 cases of GDH-HI that were confirmed by genetic diagnosis. Clinical and follow-up patient data were reviewed.

## 2. Patients and Methods

### 2.1. Subjects

A total of 240 CHI patients were investigated in Beijing Children's Hospital over the past 15 years. The diagnosis of CHI was based on diagnostic criteria described previously [1, 10, 11]. Twenty-six patients with GDH-HI were recruited into this study. Clinical information was obtained from medical records. Diazoxide was given when CHI was diagnosed. A protein-restricted diet (1–2 g/kg/d) was introduced to parents whose child was over 1 year old. The decision of whether to use the protein-restricted diet was made by the parents themselves; it was not mandatory. For the follow-up study, self-monitoring of blood glucose was required at least 2–4 times every day. The follow-up periods ranged from 6 months to 11 years. Brain development was evaluated every year at follow-up, including psychometric test, brain MRI, and EEG. Standardized psychometric tests were used to determine the cognitive, motor, speech, and social development of the patients including the Chinese version of Gesell Developmental Schedules (ages 0–6) and the Wechsler Intelligence Scale for Children (WISC) (ages 6–17). Written informed consent was obtained from parents of the proband included in the study. The study was reviewed and approved by the Beijing Children's Hospital Institutional Review Board (no. 2017-35).

### 2.2. Screening for Mutations in the *GLUD1* Gene

Genomic DNAs were extracted from the peripheral blood of probands and their parents. For patients 1, 7, 9, 12, 15, 16, 17, 18, 21, and 25, DNAs were amplified by PCR using the primer sequences described by Stanley et al. [3]. Direct Sanger sequencing of the coding region of the GLUD1 gene was performed. Exon capture was performed on DNAs of patient 2, 3, 4, 5, 6, 8, 10, 11, 13, 14, 19, 20, 22, 23, 24, and 26 using the Agilent SureSelect Human All Exon V5 Kit (Agilent Technologies Inc., Mississauga, ON, Canada). Whole-exome sequencing was performed on an Illumina HiSeq X Analyzers (Illumina, San Diego, CA, USA) using standard protocols for 150 bp paired-end runs.

### 2.3. Analysis of GDH Activity

Functional analysis was performed by analyzing the expression of wild-type and mutated GDH in 293T cells. GDH enzyme kinetics were determined spectrophotometrically in cell homogenates as we described previously [12–14].

### 2.4. Statistical Analysis

Statistical analysis was performed using SPSS for Windows, version 19.0. Data are represented as median (range).

## 3. Results

### 3.1. Clinical Manifestations

The clinical and laboratory data from the 26 patients are summarized in Table 1. None of the patients reported a family history of hypoglycemia, epilepsy, or mental retardation. The median birth weight was 3.5 kg (range: 2.9–4.5 kg) at gestational age of 37–41 weeks. Twenty-three out of 26 patients had a seizure symptom at first admission. The other three had nonspecific symptoms such as tremble, unconsciousness, and weakness. The median age of presentation was 8 months (range: 1 day to 3 years), while the median age at diagnosis was 14 months (range: 4 days to 11 years); only four patients presented with symptoms in the neonatal period. After 4–6 years old, the main clinical manifestation was postprandial hypoglycemia with protein meal. Fasting hypoglycemia was significantly reduced at follow-up.

Median serum ammonia levels were 101 *μ*mol/L (range: 37–190 *μ*mol/L). Serum ammonia concentrations were normal in 15% of patients (4/26). A high-protein diet or blood glucose level did not affect the plasma ammonia level. The plasma amino acid profile was normal in all patients. Alpha-ketoglutarate was found in urinary organic acid analysis in three out of 26 patients. Seven patients were misdiagnosed with epilepsy at the beginning, and one was misdiagnosed with a urea circulatory disorder. All patients were treated with diazoxide, with doses ranging from 2 to 12.5 mg/kg/d. The dose of diazoxide could be decreased by 1–2 mg/kg/d in the patients on protein-restricted diet (1–2 g/kg/d) compared with those without protein restriction. Some patients could maintain euglycemia using only a protein-restricted diet after the age of 6–8 years without the need for oral diazoxide. The most common side effects of diazoxide included nausea, vomiting, and loss of appetite at initial two weeks. A total of 54% (14/26) of patients had increased hair growth. Hyperammonemia is resistant to detoxification compounds (sodium benzoate and N-carbamylglutamate) or a protein-restricted diet. Serum ammonia levels remained stable during follow-up.

Psychomotor retardation was observed in 11 patients during a follow-up examination, while the remaining patients maintained a normal intelligence level. Probing the possible reason leading to psychomotor retardation, eight cases were attributed to a delayed diagnosis and the other three to poor medication compliance. Epilepsy caused by misdiagnosis or poor medication compliance was observed in 10 patients. However, patient 19 developed epilepsy at age 2 years under conditions of a timely diagnosis and ideal blood glucose control.

### 3.2. Mutation Analysis and GDH Enzyme Activity

**Figure 1:**
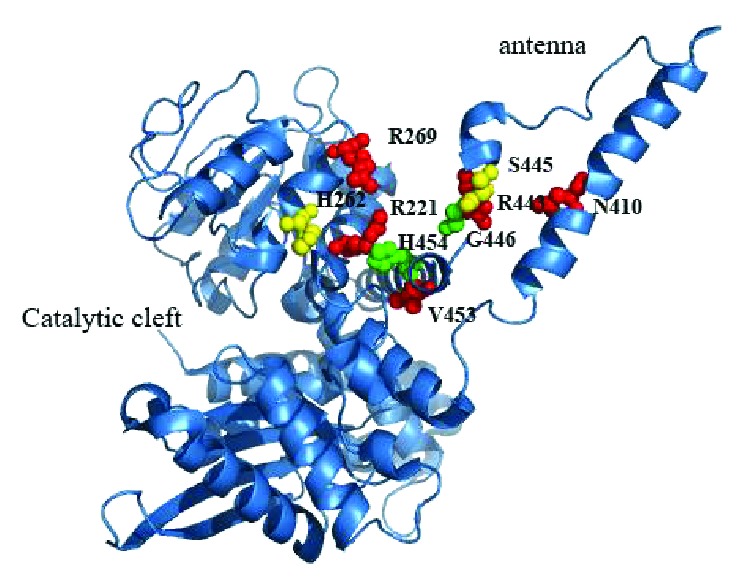
Crystal structure of human glutamate dehydrogenase (GDH) showing the location of the patient's mutations.

Based on gene sequencing results, heterozygous missense mutations in *GLUD1* were identified in all subjects (Table 1, Figure 1). Three of the mutations were observed in multiple, unrelated probands: nine had the c.1493C>T (p.S445L) mutation, six had c.965G>A (p.R269H) mutation, and four had c.820C>T (p.R221C) mutation. The mother of patient 2 and the father of patient 6 were found to carry the same mutation as their child and have mild hyperammonemia after their children were diagnosed by genetic testing. Neither of them experienced symptomatic hypoglycemia nor mental illness. Although these two patients had dominant mutations inherited from their parents, the remaining 24 probands (92%) carried sporadic de novo mutations.

As shown in Table 2, the IC50 for GTP inhibition of mutant GDH activity expressed in 293T cells was much higher than that of wild-type GDH (Table 2). Unfortunately, we were unable to correlate enzyme kinetics to the clinical phenotype in this study.

## 4. Discussion

Here, we reported 26 Chinese GDH-HI patients that were confirmed by genetic testing. The major clinical features of GDH-HI have been well characterized [12, 15]. It is typically milder and less severe than the ATP-sensitive potassium channel HI (KATP-HI). GDH-HI patients are usually appropriate for gestational age at birth. The mean age at presentation is 4 months [16], and few patients present in the neonatal period [17]. Patients do not show signs of lethargy, irritability, or coma, which are typical of other forms of hyperammonemia [18]. This serum ammonia level remained stable in follow-up and was not influenced by protein-rich meal or the onset of hypoglycemia. Efforts to lower ammonia levels in GDH-HI patients with benzoate or N-carbamoyl glutamate fail [18]. Hyperammonemia also cannot be controlled by diazoxide. In the current study, 85% (22/26) of patients exhibited a characteristic of a 3–5-fold persistent elevation in serum ammonia concentrations. All GDH-HI patients were diazoxide-responsive, and dietary protein restriction was beneficial. There was no significant correlation between the doses of diazoxide and serum ammonia levels. Ninety-two percent (24/26) of patients in the present study carried a sporadic activating mutation in *GLUD1*. GDH_S445L_ is a hotspot mutation in Chinese GDH-HI patients. The main clinical manifestations and genetic characteristics of the Chinese patients in this cohort are the same as those documented in other studies [15, 17, 19].

Although hyperammonemia is a distinguishing characteristic of GDH-HI [3, 18], the GDH_P436L_ or GDH_R269H_*GLUD1* mutations in patients with normal serum ammonia levels have been reported separately in three patients in two studies [15, 20]. In this study, four patients (15%) carrying a *GLUD1* gene mutation had normal serum ammonia levels (patients 2, 8, 10, and 23, Table 1). The proportion of patients with normal serum ammonia level is significantly higher than studies of Kapoor et al. [15] and Santer et al. [20]. To our knowledge, this is the first report of normal serum ammonia concentrations in Chinese GDH-HI patients [15, 20]. GDH-HI patients with normal serum ammonia levels who carry GDH_R221C_ and GDH_S445L_ mutations in *GLUD1* have not been described before. The possibility of postzygotic mosaicism has been reported previously, which could partially explain the normal serum ammonia levels observed [15, 18]. To investigate this hypothesis, we scrutinized our WES data from four patients and found no genotype mosaicism in circulating lymphocytes. Nevertheless, somatic mosaicism in the liver and kidney could not be excluded. There is no significant relationship between serum ammonia levels and mutation location (see Table 1). We suspect that these patients had a different genetic background involved in pathways of ammonia metabolism, which lead to different ammonia detoxification abilities. WES data from these patients and their families should be studied further. The biochemical mechanism of serum ammonia metabolism in GDH-HI needs a further study.

The frequency of epilepsy has been reported from 46% to 64% in GDH-HI patients [15, 21, 22]. Half of the patients in this study were epileptic, which is consistent with other studies [23, 24]. However, only one patient developed EEG-confirmed generalized epilepsy but a normal blood glucose level after diagnosis. The increased occurrence of epilepsy is believed to be the result of either recurrent acute brain hypoglycemia or chronic hyperammonemia injury. Another possible cause could be a decrease in the concentration of a neurotransmitter such as glutamine or *γ*-aminobutyric acid in the brain due to raised GDH activity [15]. Human glutamate dehydrogenase plays an important role in neurological diseases, tumor metabolism, and GDH-HI. In recent years, there has been growing interest in screening target inhibitors of human GDH. However, there are very few effective inhibitors of human GDH [25]. Therefore, the development of a new drug that directly targets GDH activity may confer advantages over diazoxide in terms of achieving a better prognosis for GDH-HI and reducing the incidence of epilepsy.

To date, 37 different *GLUD1* heterozygous mutations have been reported in patients with GDH-HI. These *GLUD1* mutations are located in both the catalytic (exons 6 and 7) and allosteric (exons 10, 11, and 12) domains of GDH. Only one patient with disease caused by a combination of frameshift and missense mutations that are functionally homozygous has been reported [14]. A total of 92% (24/26) of patients in the present study carried a sporadic activating mutation in *GLUD1*. These genetic characteristics are consistent with those documented in other studies [15, 17, 19, 26]. GDH_S445L_ is a hotspot mutation in Chinese GDH-HI patients, which differs from other studies [15, 27]. The mother of patient 2 and father of patient 6 were diagnosed with GDH-HI by genetic testing. Their hypoglycemia and hyperammonemia escaped recognition until their children were diagnosed. It is generally believed that GDH-HI is characterized by hyperammonemia. However, asymptomatic patients with normal blood ammonia levels were found in our study. We believe that genetic diagnosis can help us distinguish this type of patient from other types of CHI. Therefore, *GLUD1* mutational analysis may be an important tool for differential diagnosis and treatment of GDH-HI [15].

Although all mutations in this study have been reported in different studies previously, variant pathogenicities at N410S, R443W, V453M, and G446C were only predicted bioinformatically [5]. In this study, we performed GDH kinetic analysis to demonstrate the pathogenicity of all the mutations detected. In China, GDH enzymatic studies are not used regularly in clinical practice because they are expensive and time-consuming. Considering the high incidence of epilepsy, the lack of symptoms, and normal serum ammonia levels in some patients, genetic testing should be performed following a diagnosis of CHI. MacMullen et al. [17] performed enzyme kinetic studies in lymphocytes from 65 patients and found a positive relationship between median plasma ammonium and GTP IC50. Regrettably, we could not correlate enzyme kinetics to the clinical phenotype. We speculate that this may be related to using 293T cells instead of patient lymphocytes in our study. There are no clear association between clinical phenotype and mutations in this study. The number of patients in this study was relatively small considering the large population of China. As the number of patients increases, the genotype-phenotype correlation may be clear in the future.

In conclusion, the current study reported the largest Chinese cohort of GDH-HI patients. There was no significant difference between the main clinical features and gene mutations in Chinese GDH-HI patients compared with other races. Phenotypic heterogeneity was documented. A high frequency of GDH-HI with normal ammonia has been reported in this study. *GLUD1* gene analysis is helpful for treatment, follow-up, and genetic counseling because the patient may be asymptomatic or have normal ammonia concentrations. Finally, it is necessary to develop safety and specific GDH inhibitors.

## Figures and Tables

**Table 1:** Clinical and genetic characteristics of 26 GDH-HI patients.

Patient no.	Sex	Age at presentation	First clinical sign	Age at diagnosis	Birth weight (kg)	Median NH_3_ (*μ*mol/L)	Diazoxide (mg/kg/d)		Current age (y)	Psychomotor retardation		Epilepsy		Mutation	
							Initial	At last visit		Before treatment	At last visit	Before treatment	At last visit	Nucleotide	Amino acid change
1	M	4 m	Seizure	10 m	3.1	80	10	2.5	10	+	+	+	+	c.820C>T	p.R221C
2	F	1 y	Seizure	1 y and 6 m	3.7	56	7.5	5	3	−	−	−	−	c.820C>T	p.R221C
3	M	1 y	Seizure	8 y	4.5	84	2	0	10	+	+	+	+	c.820C>T	p.R221C
4	F	7 m	Seizure	2 m	3.3	91	6	4	2	−	−	−	−	c.820C>T	p.R221C
5	M	1 y	Seizure	2 y and 1 m	3.5	98	5	5	2.6	+	+	−	−	c.943C>T	p.H262Y
6	F	3 y	Seizure	11 y	3.8	103	5	5	12	+	+	+	+	c.965G>A	p.R269H
7	M	1 y	Unconsciousness	3 y	3.0	108	7.5	2.5	12	+	+	−	+	c.965G>A	p.R269H
8	M	1 y and 2 m	Trembled	1 y and 6 m	4.25	43	10	8	2.6	−	−	−	−	c.965G>A	p.R269H
9	F	1 y	Seizure	8 y	3.35	88	5	0	13.6	+	+	+	+	c.965G>A	p.R269H
10	M	9 m	Seizure	1 y and 2 m	3.6	37	5	2.5	12	−	−	−	−	c.965G>A	p.R269H
11	F	1 y	Seizure	1 y and 2 m	3.9	100	7	5	6	−	−	−	−	c.965G>A	p.R269H
12	M	6 m	Seizure	7 y	2.0	188	5	5	10	+	+	+	+	c.1388A>G	p.N410S
13	M	2 d	Seizure	6 m	3.65	106	5	0	6	−	+	−	+	c.1388A>T	p.N410I
14	M	1 m	Seizure	2 m	3.7	151	10	5	3	−	−	−	−	c.1486A>T	p.R443W
15	F	11 m	Seizure	1 y and 3 m	2.6	105	5	0	6	−	+	−	+	c.1493C>T	p.S445L
16	M	4 m	Seizure	4 m	3.2	125	7	2	10	−	−	−	−	c.1493C>T	p.S445L
17	M	6 m	Seizure	6 y	3.8	109	5	5	13	+	+	+	+	c.1493C>T	p.S445L
18	F	1 y	Seizure	1 y and 1 m	3.5	90	7.5	7.5	11	−	−	−	−	c.1493C>T	p.S445L
19	M	16 d	Seizure	2 m	3.3	161	10	7.5	2.5	−	−	−	+	c.1493C>T	p.S445L
20	F	6 d	Seizure	1 m	3.5	169	5	5	1.5	−	−	−	−	c.1493C>T	p.S445L
21	M	5 m	Seizure	5 y	4	89	5	5	10	+	+	+	+	c.1493C>T	p.S445L
22	M	8 m	Seizure	8 m	3.5	105	7.5	7.5	1.8	−	−	−	−	c.1493C>T	p.S445L
23	F	8 m	Seizure	5 m	3.65	58	5	3	2	−	−	−	−	c.1493C>T	p.S445L
24	M	1 d	Weakness	4 d	2.8	190	12.5	10	1	−	−	−	−	c.1495C>A	p.G446C
25	M	2 m	Seizure	3 m	3.2	95	7.5	5	3.9	−	−	−	−	c.1516G>A	p.V453M
26	M	8 m	Seizure	1 y and 8 m	2.8	111	8	5	3.6	−	−	−	−	c.1519G>A	p.H454Y

M: male; F: female; m: month; y: year. Normal serum ammonia level range < 74 (*μ*mol/L).

**Table 2:** Comparison of mutant and wild-type GDH enzyme kinetics in 293T cells.

293T cells	GTP IC50 (nM)
Wild type	80
p.R221C	600
p.H262Y	450
p.R269H	1060
p.N410S	53500
p.N410I	31060
p.R443W	63200
p.S445L	45100
p.G446C	48400
p.V453M	158000
p.H454Y	298000

## Data Availability

Anyone who request the data in the manuscript 2802540 can send email to Dr. Chang Su (changsulucky@yahoo.com). The clinical data, NGS data, and enzyme kinetic analysis data are fully available at any time.

## Additional Points

*Highlights*. The relationship between mutations in the *GLUD1* gene and phenotype could not be established due to phenotypic heterogeneity. Nevertheless, p.S445L mutation accounts for 35% of the mutations in GDH-HI Chinese patients. GDH-HI without hyperammonemia is more common in Chinese patients than other races.
